# Combining radiotherapy with sunitinib: lessons (to be) learned

**DOI:** 10.1007/s10456-015-9476-3

**Published:** 2015-07-23

**Authors:** Esther A. Kleibeuker, Matthijs A. ten Hooven, Henk M. Verheul, Ben J. Slotman, Victor L. Thijssen

**Affiliations:** Department of Medical Oncology, VU University Medical Center, De Boelelaan 1118, 1081 HV Amsterdam, The Netherlands; Department of Radiation Oncology, VU University Medical Center, De Boelelaan 1118, 1081 HV Amsterdam, The Netherlands

**Keywords:** Radiotherapy, Angiogenesis, Sunitinib, Combination therapy, Cancer

## Abstract

To improve the efficacy of radiotherapy (RTx), there is a growing interest in combining RTx with drugs that inhibit angiogenesis, i.e., the process of neo-vessel formation out of preexisting capillaries. A frequently used drug to inhibit angiogenesis is sunitinib (Sutent, SU11248), a receptor tyrosine kinase inhibitor that is currently FDA approved for the treatment of several cancer types. The current review presents an overview of the preclinical studies and clinical trials that combined sunitinib with RTx. We discuss the findings from preclinical and clinical observations with a focus on dose scheduling and commonly reported toxicities. In addition, the effects of combination therapy on tumor response and patient survival are described. Finally, the lessons learned from preclinical and clinical studies are summarized and opportunities and pitfalls for future clinical trials are presented.

**Electronic supplementary material:**

The online version of this article (doi:10.1007/s10456-015-9476-3) contains supplementary material, which is available to authorized users.

## Introduction

Radiotherapy (RTx) is effective against many tumor types and is used for curative and palliative purposes. Consequently, more than half of the cancer patients receive RTx [1, 2]. Despite improvements in the efficacy of this treatment modality, there are still a considerable number of patients who show tumor recurrence [1, 3]. To enhance the clinical benefit of RTx, the current research often aims to combine RTx with other treatment modalities, including angiogenesis inhibitors.

Angiogenesis is the process by which new blood vessels are formed out of preexisting vessels, and it is considered as one of the hallmarks of cancer [4]. In most tumors, an imbalance between pro- and anti-angiogenic factors exists due to tissue hypoxia. This imbalance induces the growth of an abnormally structured and leaky tumor vasculature [5]. Consequently, tissue oxygenation remains inadequate which not only causes continuous stimulation of angiogenesis but also interferes with RTx. Angiostatic drugs have been developed to counteract the imbalance between angioregulatory factors. Several of these drugs were shown to transiently induce vascular normalization in preclinical models [5]. Accordingly, the tumor perfusion briefly improved which was shown to increase the efficacy of RTx [6–8]. Whether this also occurs in human tumors is still under investigation.

**Fig. 1:**
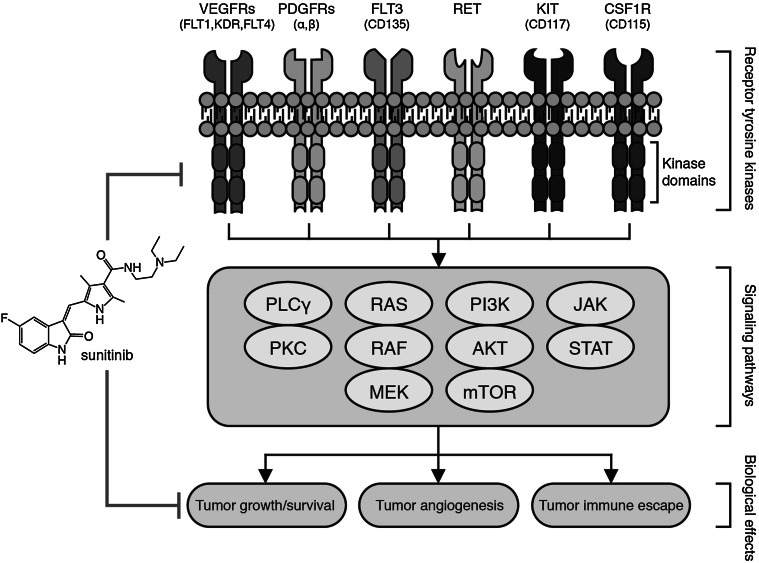
Schematic overview of the main receptor tyrosine kinases, the downstream signaling pathways, and biological processes that are targeted by sunitinib

In the last two decades, combinations of RTx with different angiostatic drugs have been evaluated [6, 9–11]. One of the frequently used drugs is sunitinib (Sutent, SU11248), a receptor tyrosine kinase inhibitor (TKI) that targets multiple receptors, including vascular endothelial growth factor receptor (VEGFR)-1, 2 and 3, platelet-derived growth factor receptor (PDGFR) *α* and *β*, stem cell growth factor (c-KIT), fms-like tyrosine kinase receptor 3 (FLT-3), neurotropic factor receptor (RET) and colony-stimulating factor (CSF-1R) [12, 13]. Binding these receptors results in the inhibition of multiple signaling pathways that are key in the growth and survival of different tumor cells as well as of endothelial cell, i.e., the cells that align a blood vessel (Fig. 1) (for excellent reviews, see [12, 14]). As a result, sunitinib acts as an effective inhibitor of tumor growth, as demonstrated in variety of xenograft tumor models. In patients, sunitinib is approved for the treatment of pancreatic neuroendocrine tumors, metastatic renal cell carcinoma (mRCC) and imatinib-resistant gastrointestinal stromal tumors. To gain better insight into the applicability of this combination therapy, we evaluated the preclinical and clinical studies that combined sunitinib with RTx (for method of the literature searches, see supplementary data). We discuss the similarities and discrepancies between preclinical and clinical observations with a focus on dose scheduling and commonly reported toxicities. In addition, the effects on tumor response and patient survival are described. Finally, the opportunities and pitfalls for future clinical trials are presented.

## Preclinical assessment of combining RTx with sunitinib

The effects of sunitinib monotherapy on angiogenesis and tumor growth are well studied and understood [12]. The effects of sunitinib in combination with RTx are less well studied, but it has been demonstrated that sunitinib given to endothelial cells (EC) before RTx enhances the apoptotic cell fraction [15, 16]. On the other hand, El Kaffas et al. [17] did not observe an enhanced effect on apoptosis. In fact, they observed that EC apoptosis was reduced when sunitinib was combined with high-dose RTx (up to 16 Gy). These discrepancies are most likely due to differences in dose scheduling emphasizing that dosing of radiation and sunitinib are important for their effects on EC apoptosis.

In tumor cells, it is generally observed that the combination therapy enhances apoptosis and reduces clonogenic survival. For example, in 4T1 breast cancer cells, the combination resulted in an increase in caspase-mediated apoptosis, while both treatments alone had no significant effect [18]. In two pancreatic adenocarcinoma cell lines (MiaPaCa2 and Panc-1), sunitinib combined with RTx decreased the activation of the Akt and Erk pathway and reduced the clonogenic survival [11]. Obviously, the responsiveness to the combination therapy depends on the presence of the receptors that are inhibited by sunitinib. This was illustrated in a study using prostate cancer cell lines lacking the target receptors in which the combination of sunitinib and RTx did not alter the clonogenic survival compared to RTx alone. The presence of at least one of the target receptors already resulted in decreased clonogenic survival during combination therapy [19]. Collectively, in vitro studies show that when combined with irradiation, sunitinib can enhance apoptosis and reduce cell survival in endothelial and tumor cells. These effects only occur when the treated cells express target receptors for sunitinib and during proper dose scheduling of both treatment modalities.

An important rationale to combine sunitinib with RTx was the observation that sunitinib can transiently improve tumor perfusion by normalizing the tumor vasculature. During this so-called normalization window, tissue oxygenation is increased which improves the efficacy of RTx. For example, dynamic contrast-enhanced (DCE) MRI analysis in a xenograft mouse model of kidney cancer revealed that improved tumor perfusion occurred after 3 days of sunitinib treatment. Applying RTx at day 3 while sunitinib treatment was continued for another 2 weeks appeared to further reduce tumor weights compared to either treatment alone although [20]. In a xenograft mouse model of squamous cell carcinoma, increased tumor oxygenation was observed after 4 days of sunitinib treatment. Applying RTx at day 4 resulted in a synergistically prolonged tumor growth delay as compared to sunitinib or RTx alone [21]. While these findings indicate that administration of sunitinib before RTx can improve therapeutic outcome due to vessel normalization, it has also been shown that simultaneous (concurrent) administration has beneficial effects on tumor growth inhibition. For example, in two studies using different xenograft models of human pancreatic adenocarcinoma, synergistic interactions on tumor growth delay were observed after concurrent treatment [11]. This could not be attributed to vascular normalization since a follow-up study using DCE-MRI showed that a decrease in *K*(trans), i.e., reduced tissue perfusion, could predict the anti-tumor effect of the combination therapy [22]. Together with observations in other xenograft models [18, 23, 24], these findings show that also concurrent sunitinib can effectively reduce tumor growth. Most likely, this is related to the increased apoptosis of EC and tumor cells as observed in the in vitro studies.

Interestingly, in a xenograft prostate cancer model, the application of sunitinib after RTx is more beneficial regarding tumor growth delay compared to concurrent sunitinib [19]. This has also been described in xenograft models of Lewis lung carcinoma (LLC) [15] and colorectal carcinoma (HT29) [25]. The mechanisms behind the beneficial effect of sunitinib treatment during or after RTx are still not fully understood but appear to be distinct from vessel normalization. A possible explanation might again be the increased apoptosis as well as the induction of cell cycle arrest and senescence by sunitinib [26]. In addition, it is also known that RTx can increase the expression of vascular growth factors, such as VEGF, thereby inducing a vascular rebound effect and tumor regrowth [27–29]. Several of these growth factors activate signaling via receptors that are inhibited by sunitinib. Consequently, sunitinib given after RTx could counteract this rebound and thus prevent tumor regrowth.

Finally, an emerging concept that might contribute to the enhanced anti-tumor effect of the combination therapy involves the immune system. While describing the mechanisms and cells involved in this response is outside the scope of the current review, both sunitinib and RTx have been shown to affect many of the cellular players involved in modulation of the immune response in the tumor microenvironment [30–37]. Consequently, it is likely that the combination of both treatment modalities influences the anti-tumor immune response. However, further research is needed to elucidate their interaction, to determine the impact of different treatment schedules and to identify which immune cells are involved.

In summary, preclinical studies show the feasibility of combining sunitinib with RTx for cancer treatment. This involves different mechanisms, including vascular normalization, modulation of cell growth and apoptosis, as well as the alterations of the immune response. A major challenge will be to translate these preclinical findings into clinically relevant treatment protocols.

## Lessons learned from combining radiotherapy with sunitinib in the clinic

Instigated by the promising results of preclinical research, several phase I and II clinical studies have been performed to assess the feasibility of combining sunitinib with RTx in cancer patients (Table 1). It should be noted that while the preclinical research aimed to elucidate the optimal scheduling, i.e., sunitinib either before, during, or after RTx, this has not been properly addressed in clinical trials. The latter studies focused more on feasibility and toxicity of the combination therapy, and in most studies, sunitinib was applied before and during RTx. Furthermore, in several studies, sunitinib maintenance therapy was an option for patients who well tolerated sunitinib treatment. Here, we focus on the two main schedules of sunitinib treatment in combination with RTx, i.e., a 6-week cycle (4 weeks on and 2 weeks off) and continuous administration.

**Table 1:** Clinical trials that evaluated the combination of RTx with sunitinib

Phase	Cancer type	Number of patients	Sunitinib				Radiotherapy		Reference
			Dose (/day)	6-week cycle/continuously	Before/concurrent/after (B/C/A) radiotherapy	Maintenance of sunitinib	Type	Dose	
1	Oligometastases	21	25–37.5–50 mg	6-week cycle	B/C	Yes: 10 patients	IGRT	40–50 Gy/10 fractions	[42]
2	Oligometastases	25	37.5 mg	6-week cycle	B/C	Yes: 9 patients	IGRT	50 Gy/10 fractions	[43]
2	mRCC	106^a^	50 mg	6-week cycle	C	Yes	SRS	Median 20 Gy per lesion	[46]
2	mRCC	22	50 mg	6-week cycle	C	No	Hypofractionated radiotherapy	Median 40 Gy/8 fractions	[47]
Case report	mRCC		50 mg	6-week cycle	A	Yes: dose reduction to 25 mg	Unkn	40 Gy/15 fractions	[48]
Case report	mRCC		50 mg	6-week cycle	B/C/A	Yes: dose reduction to 37.5 mg	Unkn	20 Gy/10 fractions	[49]
Case report	mRCC		50 mg	6-week cycle	A		Thoracic radiotherapy	Unkn	[50]
Case report	mRCC		50 mg	6-week cycle	A		WBRT	37.5 Gy/15 fractions	[51]
Case report	m ccRCC		50 mg	6-week cycle	B/A		Palliative radiotherapy	Unkn	[53]
1	Prostate cancer	17	12.5–25–37.5 mg	Continuously	B/C/A	No	External-beam IMRT	75.6 Gy/42 fractions	[56]
1	Primary CNS/mCNS tumors	15	37.5 mg	Continuously	C	Yes: 7 patients	WBRT or partial brain RT	14–70 Gy (1.8–3.5 Gy/fraction)	[64]
1/2	STS	32	50–37.5–25 mg	Continuously	B/C	No	External-beam RT	50.4 Gy/28 fractions	[63]
1	Recurrent HGG	11	37.5 mg	Continuously	C	Yes: 6 patients	Hypofractionated stereotactic RT	30–42 Gy (2.5–3.75 Gy/fraction)	[58]
2	HCC	23	25 mg	Continuously	B/C/A	Yes: 13 patients	Helical tomotherapy	Median 52.5 Gy/15 fractions	[57]
2	Non-resectable glioblastoma	12	37.5 mg	Continuously	B/C	No	Partial brain RT	60 Gy in 30 fractions	[61]
Case report	m ccRCC		Unkn	Unkn	B/A	Yes	SBRT	60 Gy/5 fractions	[52]

*m* metastatic, *RCC* renal cell carcinoma, *ccRCC* clear cell renal cell carcinoma, *CNS* central nervous system, *STS* soft tissue sarcoma, *HCC* hepatocellular carcinoma, *IGRT* image-guided radiation therapy, *SRS* stereotactic radiosurgery, *WBRT* whole-brain radiation therapy, *IMRT* intensity-modulated radiation therapy, *SBRT* stereotactic body radiation therapy

^a^45 patients sunitinib, 61 patients sorafenib

### Radiotherapy in combination with 6-week cycle sunitinib treatment

The standard administration of sunitinib is in 6-week treatment cycles with 4 weeks of 50 mg/day sunitinib and 2 weeks no treatment [12, 38]. This schedule is generally well tolerated and would allow patients to recover from the potential bone marrow toxicities [12]. The most commonly reported non-hematological adverse effects are gastrointestinal toxicities, fatigue, anorexia, hypertension, skin discoloration, and the hand-foot syndrome. Hematological toxicities include neutropenia, thrombocytopenia, anemia, and leucopenia [38–41]. In general, these adverse effects are manageable and reversible.

#### Toxicity

The main concern when combining sunitinib with RTx in patients is the possible potentiation of the frequency and severity of side effects. To address this, Kao et al. performed a dose-escalation analysis of sunitinib both before and during RTx. At the maximum tolerated dose (MTD), i.e., 10 × 5 Gy IGRT and 37.5 mg sunitinib/day, primarily grade 3 hematological toxicities were observed which were not reported as dose-limiting toxicities (DLT). Interestingly, the patients who did experience DLT had been pretreated with chemotherapy and received RTx for their liver metastases. They therefore excluded patients with liver metastasis >6 cm for their follow-up phase II trials. Although it was stated that sunitinib did not enhanced RTx toxicities, they observed that RTx enhances the hematological grade 3/4 toxicities of sunitinib [42]. In the follow-up phase II trial, the most common grade 3 side effects were again hematological, while bleeding and liver function abnormalities occurred once. Although no grade 4 side effects were observed [43], the incidence of the side effects was higher compared to studies that evaluated RTx alone [44, 45]. Relatively mild toxicity profiles, including anemia and thrombocytopenia, were also reported in two phase II trials in patients with mRCC [46, 47]. Interestingly, the side effects were not potentiated by the combination. These differences are possibly related to the tumor type or to the different RTx doses and schedules that were applied. In addition, the duration of the sunitinib treatment, i.e., single cycle versus multiple cycles, might have been of influence. For example, in two case reports in which patients received additional cycles after RTx, the patients needed dose reduction due to intolerable side effects [48, 49].

Despite the encouraging toxicity profiles, some severe toxicities incidentally occur. Tong et al. [43] reported a grade 5 gastrointestinal hemorrhage and a fatal bronchobiliary fistula, possibly related to treatment. The latter was also described in a case report in a patient who received sunitinib after thoracic RTx for a subcarinal metastasis of renal cell carcinoma [50]. Staehler et al. reported that a patient who was still on treatment with sunitinib 3 months after stereotactic radiosurgery (SRS) experienced a fatal cerebral bleeding [47]. Concerns about combining RTx with sunitinib for brain metastasis in RCC have been raised in a case report in which a patient received sunitinib after whole-brain radiotherapy [51]. Altogether, these findings show that the combination therapy is generally well tolerated, but severe complications can occur incidentally.

#### Clinical benefit

While the clinical benefit of the combination therapy has not been properly evaluated, the results from the phase I/II trials are encouraging. In patients with oligometastases, Kao et al. [42] reported complete response (CR) or partial response (PR) in 59 % of patients. Stable disease (SD) was reached in 28 % of the patients, while progressive disease (PD) occurred in the remaining patients. These response rates were favorable compared to systemic therapy alone [42]. This trial was followed by a phase II trial in a comparable patient group with 2-year follow-up [43]. The 18-month local control was 75 %, and distant control of 52 %. The median time until progression was 9.5 months, and at the end of the study, 18 patients were alive, 11 of whom without disease [43]. Encouraging results were also observed in patients with mRCC who received either sunitinib combined with single-fraction SRS [46] or high-dose hypofractionated RTx [47]. It was stated that these results were not explained by the single therapies alone which is supported by several case reports that described the beneficial effects of this combination therapy in patients with mRCC [48, 49, 52, 53]. Together, these findings demonstrate that the combination of sunitinib and RTx might induce clinical responses in different tumor types. However, a phase III clinical trial is required in order to draw firm conclusions.

Overall, the toxicities of the concurrent combination of RTx and sunitinib administered in 6-week cycles appears to depend on the duration and dose of sunitinib treatment, on the concurrent dose of RTx, but also on previous chemoradiation and type of metastases, e.g., liver or brain. Nevertheless, the combination therapy is generally well tolerated and appears to result in encouraging anti-tumor and clinical responses in a diverse range of tumors. All this warrants additional studies to further establish the clinical benefit of the combination therapy and to address the importance of dose scheduling on treatment efficacy and toxicity.

### Radiotherapy in combination with continuous sunitinib treatment

The disadvantage of interrupting the sunitinib treatment is that it potentially allows proliferation of tumor cells between the cycles. For this reason, continuous dosing of monotherapy sunitinib has also been tested. For this, the daily dose of sunitinib was reduced to 37.5 mg/day. This regimen is also well tolerated, with a similar toxicity profile compared to the 4 weeks on and 2 weeks off schedule [12, 54, 55].

#### Toxicity

Similar to the studies using a 6-week cycle treatment, the trials combining continuous sunitinib with RTx have carefully evaluated the toxicity profile. In patients with localized high-risk prostate cancer, the safe dose of continuous sunitinib in combination with external-beam RTx was determined at 25 mg/day, at which one out of six patients developed a DLT (grade 3 fatigue). The most common side effects were fatigue, neutropenia, anemia, and hypertension [56]. In a phase II study including patients with locally advanced hepatocellular carcinoma (HCC), similar common and manageable side effects were reported when continuous sunitinib treatment (25 mg/day) was combined with RTx [57]. This relatively mild toxicity profile is interesting, since all patients received RTx on the liver and, as stated before, liver irradiation appeared to be an important factor decreasing the tolerability of the sunitinib dose [42]. Possibly, the lower dose of sunitinib and the different schedules underlie the differences in the side effects. However, other factors such as tumor type and dosing of RTx could also have contributed, warranting further research.

In a phase I study in patients with primary and metastatic central nervous system malignancies, the combination of concurrent sunitinib (37.5 mg/day) and cranial RTx mainly induced manageable toxicity. The incidence and severity of the toxicities were independent of type and dose of the RTx [58]. Since the toxicity rate of the combination treatment was slightly higher compared to studies in which patients only received cranial RTx, addition of sunitinib appeared to enhance the side effects [59, 60]. In a pilot study with recurrent high-grade glioma patients, 90 % experienced grade 1/2 toxicity (mainly hematological), while only one patient had a DLT (grade 4, oral ulcer) [58]. In a following phase II study with 12 newly diagnosed, non-resectable glioblastoma patients, again the most frequently reported side effects were grade 1/2, although some grade 3 toxicities were reported [61]. However, since only two patients received the combined therapy, this should be evaluated as sunitinib monotherapy. With this in mind, sunitinib treatment was stated to be well tolerated but did not result in anti-tumor responses [61]. Comparable results were found in glioma patients who received continuous sunitinib as monotherapy prior to RTx and/or chemotherapy [62].

In contrast to the mild toxicities described so far, a phase I/II study in patients with soft tissue sarcoma was closed prematurely due to DLT when sunitinib was combined with RTx [63]. Seven patients had received 50 mg daily for 2 weeks before RTx, followed by 25 mg daily during RTx. Dose-limiting toxicities were observed in four patients (grade 3/4). Subsequently, the starting dose of sunitinib was reduced to 37.5 mg daily, followed by 37.5 mg daily during RTx. The next two patients showed DLTs (grade 3), which led to premature closure of the study. Because of the lack of clinical benefit and the majority of patients showing DLTs, the schedule and dosing of sunitinib and RTx was not recommended in this patient group [63].

Altogether, continuous dosing of sunitinib combined with RTx is generally well tolerated, although due to toxicities, a lower daily dose for sunitinib is usually required as compared to the 6-week cycle. Furthermore, for specific tumor types, this combination is not recommended as it will induce DLT and does not improve patient outcome.

#### Clinical benefit

Similar to the 6-week cycle treatment, the phase I/II trials that combine continuous sunitinib with RTx show encouraging results. A study in prostate cancer patients with a median follow-up of 19.6 months showed a median post-treatment PSA of <0.1 ng/ml. Only two out of 17 patients showed treatment failure [56]. The suggestion of clinical benefit was also reported in patients with recurrent high-grade glioma [58] as well as in patients with primary and metastatic central nervous system malignancies [64]. In the latter study, the 6-month PFS was higher compared to studies that applied cranial RTx alone for patients with brain metastasis [65, 66]. Promising clinical responses were also observed in a study with locally advanced HCC patients [57]. Interestingly, several patients continued sunitinib treatment until disease progression. The median time to progression in these patients was 10 months compared to 4 months in those who did not receive maintenance sunitinib [57]. This observation corresponds with results described in preclinical studies, where maintenance therapy was the main factor contributing to tumor growth reduction [19, 26, 67].

While several studies indicated a potential benefit of the combination therapy, less promising responses were reported in a phase II study with glioblastoma patients in which sunitinib was started 8 weeks before RTx [61]. Only 41.7 % of patients completed the 8 weeks of sunitinib prior to RTx due to tumor progression and neurological deterioration. Furthermore, none of the patients was alive after 1 year [61]. A lack of additional clinical benefit was also observed in a phase I/II study with soft tissue sarcoma patients [63].

Together, these studies demonstrate that—similar to 6-week cycle treatment—continuous sunitinib treatment combined with RTx can induce clinical responses. Also in line with 6-week cycle treatment, the response appears to depend on the tumor type and dose scheduling. Interestingly, it is suggested that mainly the maintenance sunitinib treatment contributes to better and longer disease responses.

## Future prospects: lessons to be learned

The results of the preclinical research and clinical trials have provided valuable insights into the feasibility to combine sunitinib with RTx. Furthermore, several clinical trials are ongoing (Table 2) that will further address the clinical applicability of this combination therapy. Especially with regard to dose scheduling and toxicity lessons have to be learned. Although the combination therapy appears to be well tolerated, the MTD of sunitinib depends on the scheduling that is used. Compared to the common dose for sunitinib monotherapy, i.e., 50 mg/day, the combination with RTx requires dose reductions to 37.5 mg/day in case of a 6-week cycle treatment and 25 mg/day for continuous administration [42, 43, 56, 57]. While such dose reductions generally resulted in lower toxicity rates [42, 47], there are still concerns regarding rare but severe side effects, such as perforations in the gastrointestinal tract or severe hemorrhages. Interestingly, it has been described in case reports that dose reductions do not affect tumor responses [48, 49], possibly because sunitinib is known to accumulate in the tumor [25]. This is also supported by our recent preclinical study in which sunitinib dose reductions of 50 % did not affect the tumor growth delay in combination with RTx [67]. Dose reduction of sunitinib would not only reduce the severity and frequency of side effects, but also lower the financial burden on the healthcare system [68]. Therefore, future research should further resolve whether low-dose sunitinib treatment, i.e., dosing below the MTD, would affect the response rates in patients. Measurements of tumor perfusion during treatment could be of value to get better insight into the dose–response relationship. Regarding this, an ongoing phase I study (Table 2, NCT01308034) performs DCE-ultrasonography (DCE-US) after start of sunitinib to measure neo-angiogenesis. These data can provide valuable insights into the dose-dependent intra-tumoral effects of sunitinib on perfusion and angiogenesis.

**Table 2:** Ongoing clinical trials

NCT number + status	Phase	Cancer type	Sunitinib				Radiotherapy		Neo/adjuvant (N/A) to surgery	Additional drug therapy
			Dose (/day)	Cycle/continuously	Before/concurrent/after (B/C/A) radiotherapy	Maintenance of sunitinib	Type	Dose		
NCT01498835 unknown	1	LA or recurrent STS	25–37.5 mg	Continuously	B/C	No	IMRT	50.4 Gy in 28 fractions	N	–
NCT01308034 recruiting	1	Non-resectable non-GIST sarcoma	25–37.5–50 mg	Continuously	C	No	Unkn	Daily fractions over 6 weeks	–	–
NCT00437372 completed	1b	HNC, pelvic cancer, CNS tumors, thoracic neoplasms	Unkn	Unkn	C	No	EBRT	5 Fractions/week over max 8 weeks	–	–
NCT00906360 terminated	1	LA or recurrent HNSCC	Unkn	Continuously	C	No	3D-CRT	Daily fractions over 7–9 weeks	–	Cetuximab
NCT00981890 recruiting	1	Brain metastases	Unkn	Continuously	B/C/A	Yes	SRS	1 Fraction	–	–
NCT00463060 unknown	1/2	Oligometastatic disease	Unkn	Unkn	C	Unkn	Unkn	Unkn	–	–
NCT00631527 completed	1	High risk and LA Prostate cancer	≥12.5 mg	Continuously	B/C	No	Unkn	5 fractions/week over max 8 weeks	–	Hormone therapy
NCT00734851 ongoing, not recruiting	2	Prostate cancer	37.5 mg	2 weeks on, 1 week off	B	No	EBRT	66 Gy over 6–7 weeks	–	Docetaxel prednisone
NCT00400114 ongoing, not recruiting	2	Resectable esophageal cancer	12.5–50 mg	Unkn	A	Yes	Unkn	50 Gy over 4–9 weeks	A	Irinotecan, cisplatin
NCT00570908 terminated	2	CNS metastases from breast cancer	37.5 mg	Unkn	A	Yes	WBRT	30 Gy in 10 fractions	–	Capecitabine
NCT01100177 completed	2	Newly diagnosed GBM	37.5 mg	Continuously	B/C/A	Yes	Unkn	60 Gy in 30 fractions	–	–
NCT02019576 recruiting	2	m ccRCC	First-line systemic dose	6-week cycle	C	Yes	SRT	15–60 Gy in 1–8 fractions	–	–

*GBM* glioblastoma, *STS* soft tissue sarcoma, *HNC* head and neck cancer, *HNSCC* head and neck squamous cell carcinoma, *LA* locally advanced, m* metastatic, *ccRCC* clear cell renal cell carcinoma, *CNS* central nervous system, *HCC* hepatocellular carcinoma, *3D* three dimensional, *CRT* conformal radiation therapy, *SRT* stereotactic radiation therapy, *EBRT* external-beam radiation therapy, *IGRT* image-guided radiation therapy, *SRS* stereotactic radiosurgery, *WBRT* whole-brain radiation therapy, *IMRT* intensity-modulated radiation therapy, *SBRT* stereotactic body radiation therapy, *Unkn* unknown, – not applied

Another important lesson to be learned concerns the proper scheduling of both treatment modalities. Sunitinib treatment is often applied several weeks before RTx. This might be beneficial since sunitinib treatment has been shown to induce transient vascular normalization in preclinical models, resulting in improved tumor oxygenation [20, 21, 69]. However, evidence for such a response in patients should be addressed by future trials, for example with perfusion measurements using DCE-MRI [70–72] or by hypoxia imaging techniques such as FMISO PET [73, 74]. On the other hand, in the preclinical models, vascular normalization occurs rapidly after the start of treatment and lasts for only a few days. This suggests that even when vascular normalization occurs in the clinical setting, the window of opportunity has already passed when sunitinib treatment is given for several weeks prior to RTx. This is supported by a study of Lewin et al. [63] where DCE-MRI and FAZA-PET/CT analyses showed decreased tumor perfusion and increased tumor hypoxia after 2 weeks of sunitinib.

While the clinical benefit of sunitinib treatment prior to RTx is still unclear, there is ample preclinical evidence supporting a beneficial role of sunitinib maintenance therapy after RTx [15, 19, 57]. The mechanisms responsible for this are poorly understood but appear to be distinct from vessel normalization. Possibly, sunitinib counteracts the vascular rebound effect induced by RTx or improves the anti-tumor immune response. Unraveling these mechanisms requires further research. Furthermore, most clinical trials in which patients received maintenance sunitinib did not report on differences in tumor response rates or survival compared to patients who did not continue sunitinib treatment [42, 43, 46, 64]. This provides an opportunity for future research, and several ongoing studies have included sunitinib treatment after RTx (Table 2). These studies might give more insight into the potentially favorable effect of sunitinib maintenance therapy.

Another unexplored area in scheduling is the interaction between both treatment modalities when sunitinib has been part of a previous treatment regime. It has not been established whether RTx can be applied safely after long-term sunitinib treatment, whether sunitinib treatment has to be discontinued, or whether continuation improves tumor outcome. It has been shown in mRCC patients that discontinuation of sunitinib rapidly results in an angiogenic rebound [75]. Whether this happens in other tumor types as well and how this affects the efficacy and toxicity of subsequent RTx should be further addressed.

Of note, while the current review is focused on combining sunitinib with RTx, many of the future challenges reported here for sunitinib, also apply to other angiogenesis inhibitors. Differences in dose scheduling, type of drug, and tumor type will influence the therapeutic efficacy [76]. For example, the combination of bevacizumab (anti-VEGF antibody) and RTx can induce encouraging response rates [77, 78] or increased toxicity without any response [79, 80]. Similar divergent responses have been described for the combination of RTx with sorafenib, a TKI that targets several angiogenesis-related proteins, including VEGFR, PDGFR, and Raf kinases [81–83]. Unraveling the similarities and differences when combining angiostatic drugs with RTx requires a more systematic preclinical and clinical approach including, for example, imaging techniques to measure perfusion and early tumor responses [84].

In conclusion, the combination of sunitinib and RTx is a promising treatment strategy which deserves further preclinical and clinical investigation. Given the observed increased side effects of this combination therapy, research should focus on determining the maximum effective dose of sunitinib as well as on deciphering the optimal treatment schedules of the combination therapy. With all the lessons learned and lessons to be learned, the translation of the insights from phase I/II clinical trials into clinical phase III trials will reveal whether this combination therapy is really beneficial and could be implemented in daily clinical practice.

## Electronic supplementary material

Supplementary material 1 (DOCX 10 kb)
