# Supplementary material for: Combining radiotherapy with sunitinib: lessons (to be) learned
Source: Angiogenesis. 2015 Jul 23;18(4):385–95. doi: 10.1007/s10456-015-9476-3 (PMC4596900; doi:10.1007/s10456-015-9476-3)
Supplement: Supplementary file 1 — Supplementary material 1 (DOCX 10 kb) [file 10456_2015_9476_MOESM1_ESM.docx]

**Supplementary data**

**Method of literature search**

The evaluated studies were selected out of the PubMed and Embase databases by performing the following search: sunitinib [supplementary concept] OR sunitinib [tiab] AND radiotherapy [supplementary concept] OR radiotherapy [tiab]. Also, all synonyms for sunitinib and RTx were included in the search. This resulted in a total of 133 studies that were subsequently manually screened to select only the studies that combined sunitinib and RTx or studies in which sunitinib was used in patients that were previously treated with radiotherapy.

The ongoing clinical trials were selected from clinicaltrials.gov and the only selection criterion was that patients had to receive both sunitinib and RTx.
